# Local Moment Instability of Os in Honeycomb Li_2.15_Os_0.85_O_3_

**DOI:** 10.1038/s41598-018-25028-0

**Published:** 2018-04-26

**Authors:** M. K. Wallace, P. G. LaBarre, Jun Li, S.-T. Pi, W. E. Pickett, D. S. Dessau, D. Haskel, A. P. Ramirez, M. A. Subramanian

**Affiliations:** 1 Department of Chemistry, Oregon State University, Corvallis, OR 97331 USA; 2 Department of Physics, University of California Santa Cruz, Santa Cruz, CA 95064 USA; 3 Department of Physics, University of California Davis, Davis, CA 95616 USA; 4 Department of Physics, University of Colorado, Denver, CO 80309 USA; 5 Advanced Photon Source, Argonne National Laboratory, Argonne, IL 60439 USA

## Abstract

Compounds with honeycomb structures occupied by strong spin orbit coupled (SOC) moments are considered to be candidate Kitaev quantum spin liquids. Here we present the first example of Os on a honeycomb structure, Li_2.15(3)_Os_0.85(3)_O_3_ (*C*2/*c*, *a* = 5.09 Å, *b* = 8.81 Å, *c* = 9.83 Å, *β* = 99.3°). Neutron diffraction shows large site disorder in the honeycomb layer and X-ray absorption spectroscopy indicates a valence state of Os (4.7 ± 0.2), consistent with the nominal concentration. We observe a transport band gap of Δ = 243 ± 23 meV, a large van Vleck susceptibility, and an effective moment of 0.85 *μ*_B_, much lower than expected from 70% Os(+5). No evidence of long range order is found above 0.10 K but a spin glass-like peak in ac-susceptibility is observed at 0.5 K. The specific heat displays an impurity spin contribution in addition to a power law ∝T^(0.63±0.06)^. Applied density functional theory (DFT) leads to a reduced moment, suggesting incipient itineracy of the valence electrons, and finding evidence that Li over stoichiometry leads to Os(4+)−Os(5+) mixed valence. This local picture is discussed in light of the site disorder and a possible underlying quantum spin liquid state.

## Introduction

**Figure 1:**
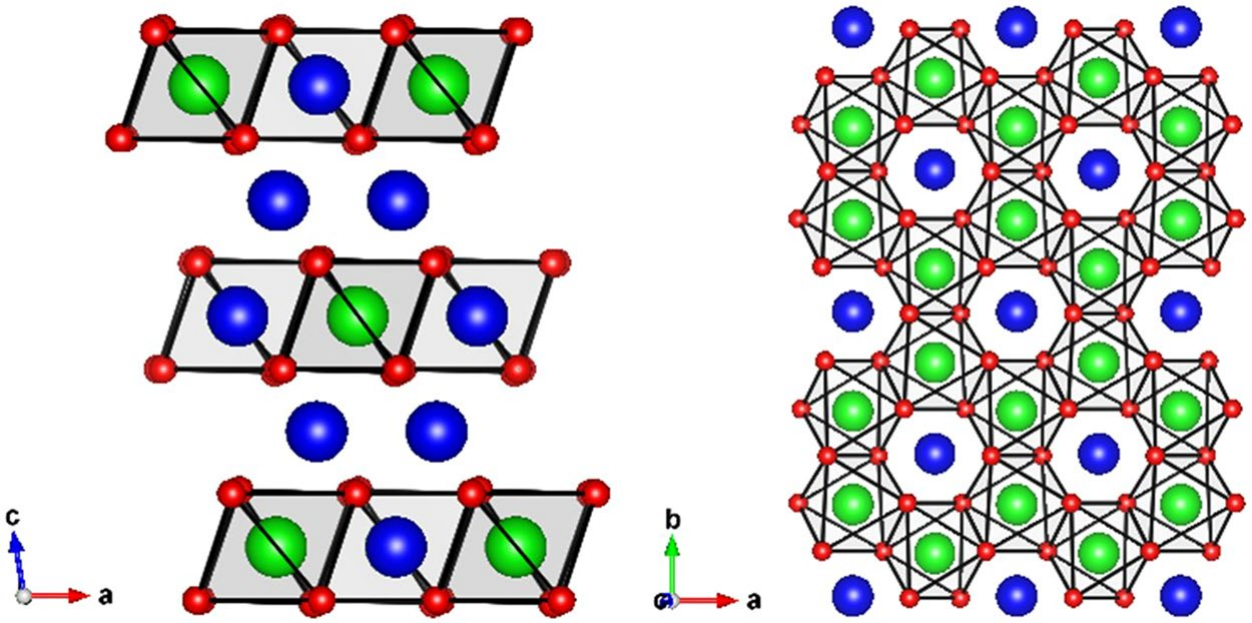
Left: Li_2_MO_3_ (M = 4d or 5d element) structure as alternating Li and LiM_2_ layers. Right: Viewed along the c axis the LiM_2_ layer, where MO_6_ edge-sharing octahedra form the honeycomb lattice. Blue, green, and red spheres represent lithium, metal, and oxygen atoms respectively.

The demonstration by Kitaev of an exactly solvable quantum spin liquid (QSL) model incorporating S = $\frac{1}{2}$ spins with anisotropic interactions on a honeycomb lattice^1,2^ has motivated searches for its experimental realization. Whereas magnetic honeycomb-containing compounds have been extensively investigated in 3d and 4d metal oxides^3–11^, the strong interaction anisotropy required by Kitaev’s theory has placed a focus on 5d metal oxides, for which strong spin-orbit coupling (SOC), the origin of spatial anisotropy, can be expected^12–18^. In the A_2_MO_3_ honeycomb structure, where A is an alkali element and M a 4d or 5d element, the AM_2_ layers form a hexagonal network of edge sharing MO_6_ octahedra with a single A^+^ ion at the centers of the hexagons (Fig. 1).

Examples such as $\alpha$-Na_2_IrO_3_ and $\alpha$-Li_2_IrO_3_ with effective spin *J*_*eff*_ = $\frac{1}{2}$, have recently emerged as possible examples of Kitaev physics. These compounds possess antiferromagnetic (AF) Weiss temperatures of 125(6) K and 33(3) K and undergo AF order at 15.5 K and 14.5 K for Na_2_IrO_3_ and Li_2_IrO_3_ respectively^19,20^. The 3D hyper-honeycomb lattice compound $\beta$-Li_2_IrO_3_, however, demonstrates a ferromagnetic (FM) Weiss temperature of 40 K and weak ordering signatures at 38 K among *J*_*eff*_ = $\frac{1}{2}$ moments^21^. Among non-oxide materials, the layered compound $\alpha$ -RuCl_3_ has also been discussed as a possible Kitaev system, though it too undergoes long range order at 7.5 K^22^. Since the suppression of classical order via geometrical frustration is a requirement for creating a QSL state, the above systems, while possessing important attributes, fall short of the Kitaev criteria^23^. Due to the ordering seen in other honeycomb compounds and because the specific materials conditions required to produce a QSL are ill-defined at present, it is important to study other honeycomb-containing compounds with sizable SOC.

Here, we ask what would be the result of *reducing* the SOC from that of 540 meV in Ir to 480 meV in Os^24^. In the present work we report on the synthesis, structure, and properties of Li_2.15(3)_Os_0.85(3)_O_3_ which is isostructural to the Ir honeycomb compounds mentioned above. For the stoichiometric compound, Li_2_OsO_3_, the Os ion is expected to be in the 4+ = d^4^, *J*_*eff*_ = 0 state to maintain charge-neutrality. In our work, we find, in contrast to other honeycomb systems, a lack of long range order above 0.1 K. While this might be due to a frustrated lattice, it also might be due to site disorder among the Os ions. For each crystallographic site representing the LiOs_2_ layer, an average of 43% of the sites (compared to the expected 33 $\%$ of these sites) are occupied by lithium and thus the average valence of Os is +4.5 $\pm$ 0.1 (d^3.5^), a value consistent with our X-ray absorption spectroscopy (XAS) measurements, which yield +4.7 $\pm$ 0.2. From a local moment perspective and using the XAS-determined valence state, our system might be comprised of 30% d^4^ (*J*_*eff*_ = 0) and 70% d^5^ (*J*_*eff*_ = $\frac{3}{2}$) but its physical properties are not easily understood. We find a transport gap of 243 meV, an effective magnetic moment of 0.85 *μ*_B_, which is much less than the effective moment of 3.24 *μ*_B_ expected if 70 $\%$ of the ions possessed *J*_*eff*_ = $\frac{3}{2}$. No magnetic order is observed above 0.10 K, and the specific heat obeys a fractional power law in temperature and is only weakly magnetic field-dependent. We discuss the constraints on the local physics of Os from a band structure perspective and their implications for collective behavior en route to a possible QSL in SOC honeycomb systems.

## Results and Discussion

### Structure

Common space groups assigned to honeycomb-structure compounds Li_2_MO_3_ (M = Mo, Mn, Rh, Ir, Ru, Pt, and Sn) are *C*2/*m*, *C*2/*c*, and *R*$\bar{3}$*m*^4,7–9,11,17,25–32^. Supplemental Figure S1 represents Li_2_MO_3_ *C*2/*m*, *C*2/*c*, and *R*$\bar{3}$*m* unit cells illustrated down the b-axis (top) and corresponding portion of the Li-M layer representing a (bottom) with lithium and metal occupying their ideal Wyckoff positions^4,11,17,28,29,32^. For all three space groups, edge sharing octahedral LiM_2_ layers alternate with edge sharing octahedral Li layers. The difference in space groups and their associated symmetries can in part be ascribed to stacking of the LiM_2_ layers, with *R*$\bar{3}$*m* as the highest in symmetry. The space group assignments of some Li_2_MO_3_ systems has been controversial. For example, Li_2_MnO_3_ was first refined to be *C*2/*c*, but later found to be *C*2/*m* on the basis of electron diffraction and transmission electron microscopy^26,27^.

**Figure 2:**
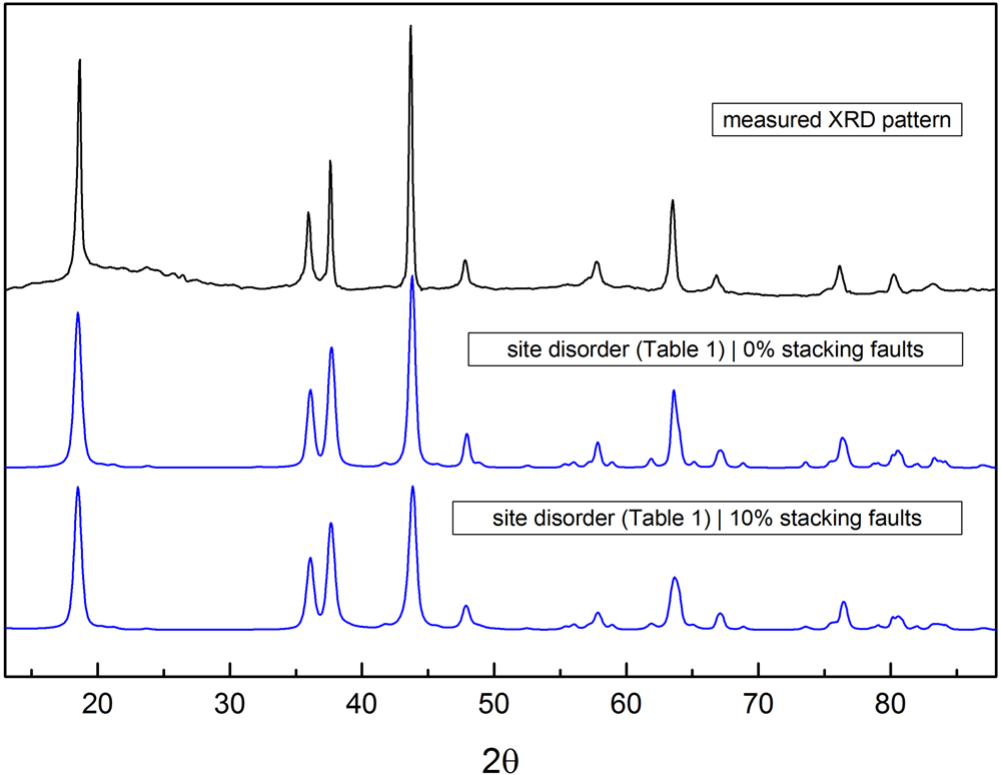
A comparison of the measured XRD pattern (top black line) (*λ* = 1.514 Angstroms), along with 43$\%$ site disorder (Table 1) $|$0$\%$ stacking fault and 43$\%$ site disorder (Table 1) $|$10$\%$ stacking fault simulated DIFFaX patterns (blue lines).

Another example is Li_2_MoO_3_, which was reported as both *C*2/*c* and *R*$\bar{3}$*m*^4,28^. When comparing literature on polycrystalline Li_2_IrO_3_ synthesized under standard solid state conditions, discrepancies in *C*2/*c* and *C*2/*m* space groups exist. Recent work supports the higher symmetry *C*2/*m* as the appropriate space group for polycrystalline Li_2_IrO_3_^17,29,32^. The powder X-ray diffraction pattern of Li_2_OsO_3_ is shown in Fig. 2 (top black line).

At first glance, the X-ray pattern pointed to a more symmetric space group, *R*$\bar{3}$*m*, however, close examination of the pattern showed weak and broad diffraction peaks in the 2$\theta$ region of 19° to 33°. It is known that for both *C*2/*m* and *C*2/*c* systems, with no Li-M site exchange within the LiM_2_ layers, sharp peaks exist within the 19° to 33° range. However, introducing Li-M site exchange within the LiM_2_ layers decreases the relative intensities of these peaks, leading to virtually no peak presence at 30$\%$ Li-M site exchange^30^. Such a reduction in diffraction peak intensity is also found for (hk0) reflections past 38° ^30^. Disorder among Li-M sites is commonly reported for honeycomb layered metal oxides since all corresponding crystallographic sites are octahedral and similar in size^4,9,11,17,18,26–28^. The presence of stacking faults associated with a shift between successive LiM_2_ layers will cause the peaks in the region from 19° to 33° to further broaden asymmetrically^7–9,18,30^. Thus, with the existence of Li-M site disorder and stacking faults, it is difficult to distinguish between the corresponding space groups using powder X-ray diffraction. The x-ray scattering length for lithium and oxygen are also small due to their low *Z*. Because of the neutron scattering lengths of lithium, osmium, and oxygen, neutron diffraction is ideal to characterize the Li_2_OsO_3_ structure.

**Figure 3:**
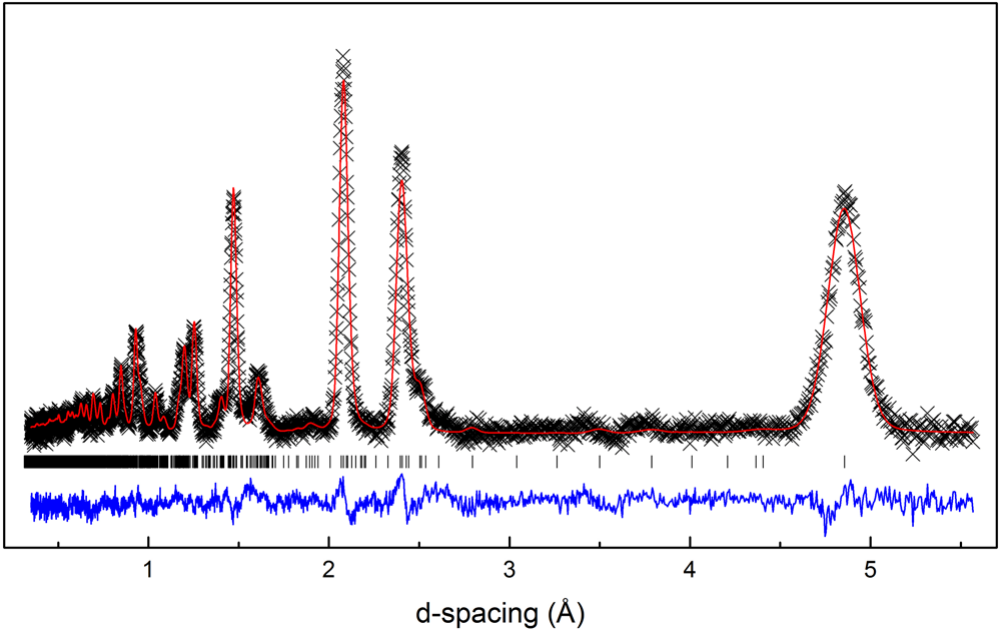
Rietveld refinement of TOF Neutron (Oak Ridge NOMAD BL-1B) diffraction data. The collected data (black cross), Rietveld refinement (red line), and difference (blue line) are presented for one of the four collected banks. Resulting cumulative *wR*_p_ = 6.4 $\%$.

To determine the crystal structure, Rietveld refinements were performed on room temperature neutron diffraction data using the GSAS program (Fig. 3)^33,34^. The current synthesis procedure restricted diffraction measurements to the Oak Ridge NOMAD TOF Neutron beamline, which is well-suited for small sample sizes. A pseudo-Voigt peak shape profile was chosen and parameters refined to obtain the best fit to the collected data. The space group was refined to be *C*2/*c*, with lattice dimensions *a* = 5.09 Å, *b* = 8.81 Å, *c* = 9.83 Å, and *β* = 99.3°. Rietveld refinements for all collected banks are shown in Supplemental Figure S2, with cumulative *wR*_p_ = 6.40 %. The atomic coordinates, occupancies, and isotropic displacement parameters are represented in Table 1.

**Table 1:** Atomic coordinates, occupancies and isotropic displacement parameters obtained from Rietveld refinement (*C*2/*c*) of TOF Neutron (Oak Ridge NOMAD BL-1B) diffraction data.

	Wyckoff	*x*	*y*	*z*	*occ*	*U*_*iso*_
Li1	8*f*	0.2543(2)	0.0840(1)	0.0044(9)	1	0.25(8)
Li2	4*d*	0.25	0.25	0.5	1	0.037(4)
Li3	4*e*	0	0.7531(8)	0.25	0.32(4)	0.20(7)
Os3	4*e*	0	0.7531(8)	0.25	0.68(4)	0.20(7)
Os4	4*e*	0	0.0777(7)	0.25	0.46(2)	0.0043(2)
Li4	4*e*	0	0.0777(7)	0.25	0.54(2)	0.0043(2)
Os5	4*e*	0	0.4026(6)	0.25	0.56(2)	0.0082(2)
Li5	4*e*	0	0.4026(6)	0.25	0.44(2)	0.0082(2)
O1	8*f*	0.1335(7)	0.2414(5)	0.1344(4)	1	0.029(3)
O2	8*f*	0.1269(8)	0.5770(5)	0.1414(4)	1	0.0085(3)
O3	8*f*	0.1387(8)	0.9181(5)	0.1332(5)	1	0.0082(8)

Interatomic distances and angles are given in Supplemental Table S1. No osmium is detected in the lithium-only layers. For *C*2/*c* there are three unique atomic positions to describe the Li and Os sites within the LiOs_2_ layer (Supplemental Figure S3). Shown in Table 1, corresponding sites are labeled as Li/Os 3, 4, and 5. If no Li-Os site disorder existed within the LiOs_2_ layer, only Li3, Os4, and Os5 would exist (each with an occupancy of 1), with Os4 and Os5 sites describing the honeycomb rings. As shown from Table 1 occupancies, a large percentage of Li-Os site disorder exists within the LiOs_2_ layer. For each of the three respective crystallographic sites, an average of 43$\%$ is occupied by lithium. The stoichiometry derived from occupancy refinements of Li, Os, and O is Li_2_._15(3)_Os_0.85(3)_O_3_ suggesting an average osmium oxidation state of 4.5 $\pm$ 0.1. It is important to note that oxygen occupancy refinements indicate that the oxygen sites are fully occupied and no detectable vacancies are observed. Though 2:1 stoichiometric amounts of Li:Os were used in the synthesis, the refined stoichiometric ratio is considered reasonable as the presence of a small amount of osmium impurity is formed during the synthesis and was removed by heating the sample in air at 300 °C as OsO_4_ through sublimation.

Stacking faults were modeled using the DIFFaX program^35^ and the description of the model and analogous XRD patterns are discussed in the SI section. From the discussion presented, the absence of the (020) peak can only be attributed to Li-Os site disorder within the LiOs_2_ layers and not from stacking faults, consistent with the neutron refinement. As shown in Fig. 2, the measured XRD pattern is compared to simulated DIFFaX patterns with site disorder representing Table 1 refined Wyckoff site occupancies with and without 10$\%$ stacking faults (blue lines).

**Figure 4:**
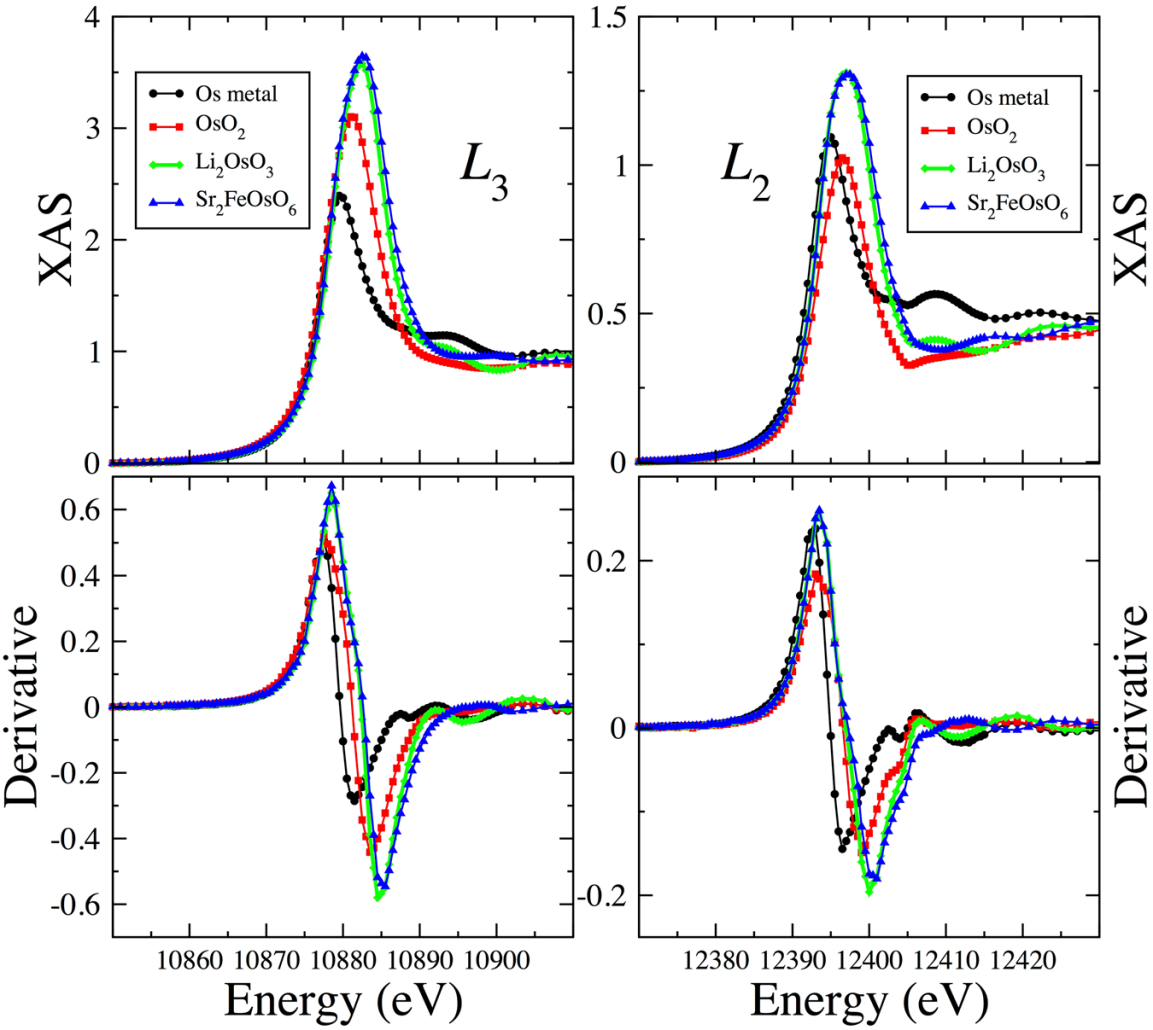
XAS measurements at the Os *L*_*2,3*_ absorption edges on Li_2.15_Os_0.85_O_3_ and three reference compounds with known oxidation state.

XAS measurements at the Os *L*_*2*,3_ absorption edges ($2{{\rm{p}}}_{\frac{1}{2},\frac{3}{2}}$ → 5d resonant excitation) were used to provide an additional estimate of Os valence (Fig. 4). The enhanced X-ray absorption above the leading edge (“white line”) is a result of large density of empty 5d states near the Fermi level. This peak grows in intensity and shifts to higher energy with increasing Os oxidation state. Interpolating the XAS peak position in the honeycomb sample onto those of the reference compounds yields an oxidation state of +4.7 $\pm$ 0.2, within errors of results from structure refinements.

The ratio of *L*_*3*_ to *L*_2_ white line intensity, also known as the isotropic branching ratio (BR), provides a measure of the relevance of SOC interactions in the 5d band^36,37^. In the absence of sizable SOC interactions, the isotropic branching ratio equals 2 reflecting the different occupancies of the core levels at *L*_3_ and *L*_2_ edges. We measured BR = 2.9(1) which significantly differs from the statistical value of 2 and indicates that SOC interactions need to be included in order to describe the 5d electronic structure of this compound.

### Thermodynamic and Transport Properties

**Figure 5:**
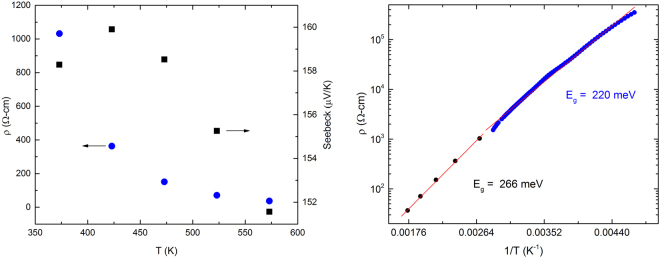
Left: High temperature resistivity and Seebeck coefficient data of Li_2.15_Os_0.85_O_3_ sample from 350 to 600 K. Right: Resistivity versus temperature for Li_2.15_Os_0.85_O_3_ in the range 200–600 K versus inverse temperature. The low and high temperature measurements were performed in different apparatuses on samples from the same growth run. To assure continuity at T = 300 K, the data have been rescaled for the low temperature measurement due to its greater uncertainty in the geometric factor.

Temperature dependent resistivity and Seebeck measurements from 300–600 Kelvin are shown in Fig. 5. The gap energy (*E*_*g*_) for the sample was extracted using $\rho$ = ${\rho }_{o}$exp(*E*_*g*_/2*k*_*B*_*T*) with *E*_*g*_ = 220 meV and 266 meV obtained from low- and high-temperature transport measurements respectively, thus indicating a small band gap insulator (Fig. 5 - right).

**Figure 6:**
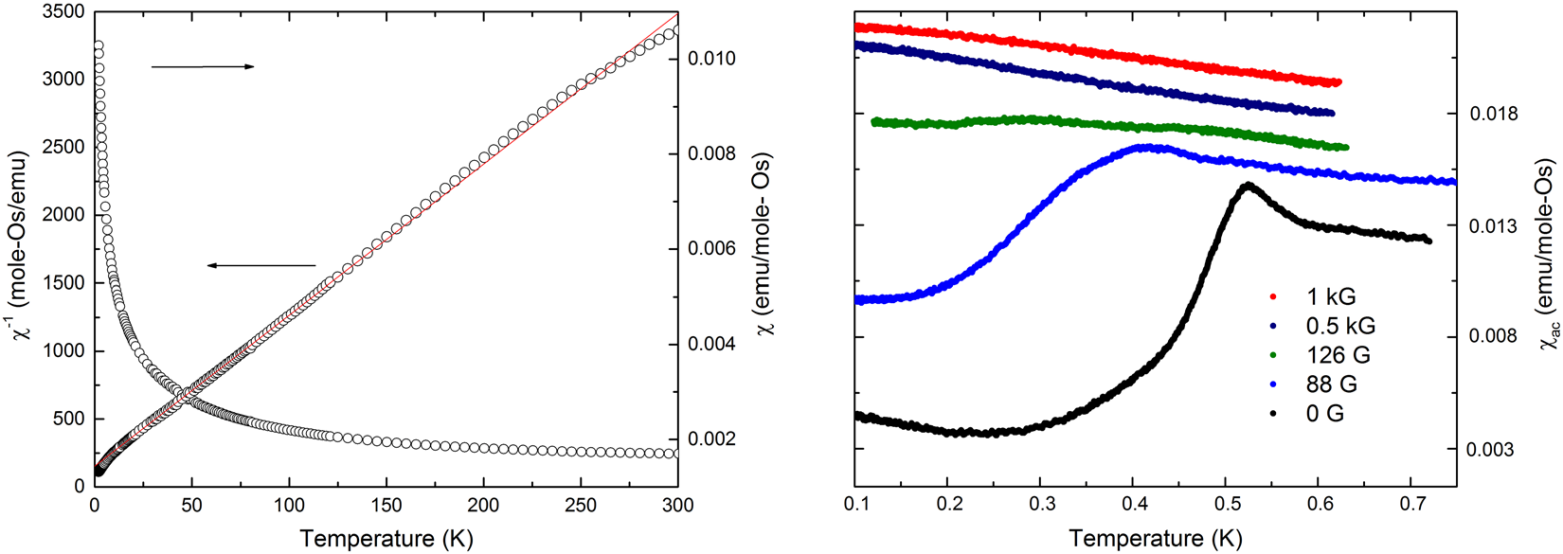
Left: DC-Susceptibility Li_2.15_Os_0.85_O_3_ versus temperature. Also shown is the inverse susceptibility after subtracting a van Vleck term as described in the text. Right: ac-susceptibility versus temperature at different values of applied magnetic field. The curves have been offset vertically by 0.0020, 0.0032, 0.0044, and 0.0056 emu for *H* = 88, 126, 500, and 1000 G respectively, for clarity.

The magnetic susceptibility, ${\chi }_{dc}$, for *H* = 0.5 T (Fig. 6) suggests the combined effects of Curie-Weiss as well as van Vleck temperature independent paramagnetism over the entire measurement range above 2 K. Within this assumption, we varied the magnitude of the van Vleck term, *χ*_*VV*_, to produce a pure Curie-Weiss contribution. We found that subtractin ${\chi }_{VV}$ = 0.00135 emu/mole from the measured $\chi$(*T*) produces the straightest 1/*χ*(*T*), resulting in a good fit to the Curie-Weiss form, *χ* = *C*/(*T − θ*), where *C* is the Curie constant and, $\theta$ is the Weiss constant.

Fitting the data between 50 and 200 K yields a Weiss constant of *θ* = −11.5 K and a finite effective moment $\mu$_*eff*_ = 0.85 ${\mu }_{B}$, significantly greater than expected for the *J*_*eff*_ = 0 state of Os(+4). Given the above XAS and neutron scattering refinement results showing that the average Os valence is 4.7, however, an alternate ionic scenario is for 70$\%$ of the Os ions to be in the +5 state (*J*_*eff*_ = $\frac{3}{2}$) and 30% in the +4 state (*J*_*eff*_ = 0). This analysis yields ${\mu }_{{\rm{eff}}}$ = 1.01 ${\mu }_{B}$ (*θ* = −11.8 K) for the magnetic (+5) ions, which is now significantly less than the expected moment.

We discuss possible sources of this discrepancy below. These *χ*_*dc*_ data were augmented with ac-susceptibility (*χ*_*ac*_) data down to 0.1 K, which were calibrated to the *χ*_*dc*_ data in the overlapping temperature range 2.0–2.5 K. A peak in *χ*_*ac*_ is observed at 0.5 K, but is rapidly suppressed by magnetic fields far less than 0.1 T. Given the usual relationship between *H* and T for a g-factor of two, one expects suppression of an antiferromagnetic ordering feature at 0.5 K for *H* values an order of magnitude larger than observed. Alternatively, such a cusp in *χ*_*ac*_ can be attributed to spin glass freezing, a scenario consistent with the high degree of disorder in this spin system. The spins involved in such freezing may not represent the bulk of the Os(+5) spin population, as we argue below.

**Figure 7:**
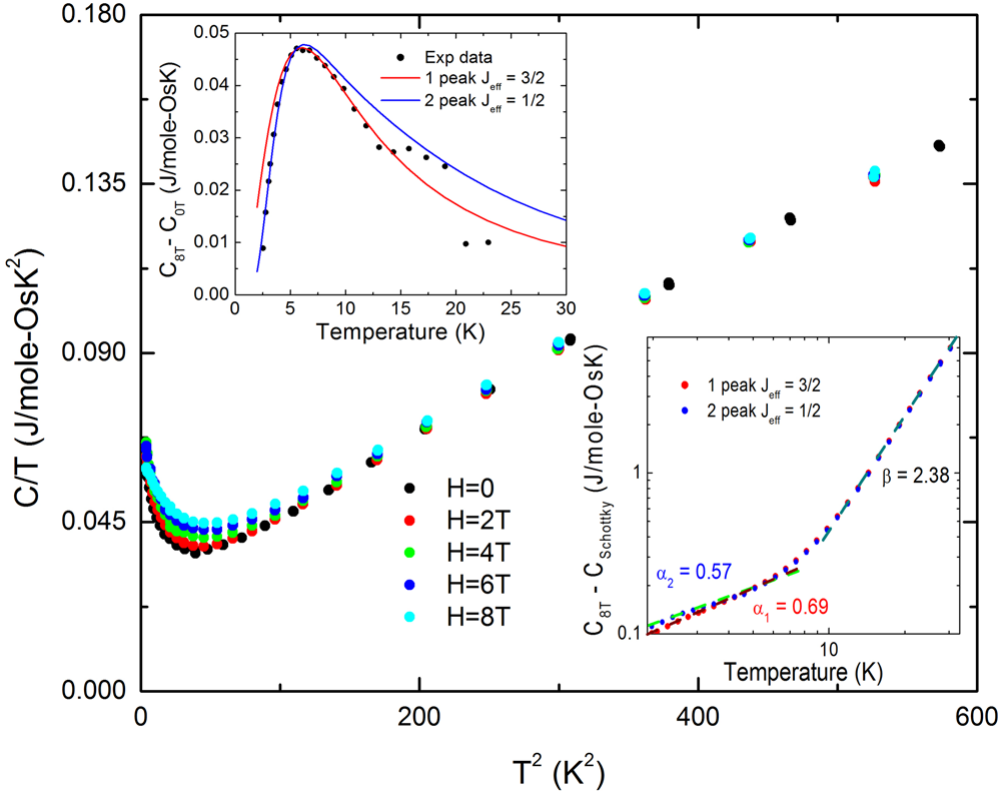
The specific heat C(T) of Li_2.15_Os_0.85_O_3_ in various applied fields, measured using a Physical Property Measurement System. Upper Inset: Impurity spins modeled with a Schottky anomaly. Lower Inset: Specific heat after removing the impurity contribution.

The existence of a small subset of spins that are interacting at a mean field energy scale of k_B_T for T = 0.5 K, as suggested by the *χ*_*ac*_ peak, is also supported by *C*(*T*, *H*), shown in Fig. 7. Here, *C*/*T* exhibits an upturn below its minimum at T = 6 K. This upturn is only moderately affected by fields up to 8 T, so we model this as *C* = *C*_1_(*T*) + *C*_2_(*T*, *H*). Here *C*_1_ is a combination of the lattice specific heat and an H-independent electronic contribution. Taking the difference between *C*(*H* = 8 T) and *C*(*H* = 0), we find that *C*_2_(*T*, *H* = 8 T) resembles a broadened Schottky anomaly (Fig. 7 upper inset). We can fit this contribution to either a single *J*_*eff*_ = $\frac{3}{2}$ (g = 1.3) Schottky anomaly or a pair of *J*_*eff*_ = $\frac{1}{2}$ (g = 2.6 and 7.4) Schottky anomalies with molar concentrations of 4.1$\%$ and 9.6$\%$ (total for the pair) respectively (Assuming 70 $\%$ of the spins are magnetic, these fractions become 5.9$\%$ and 13.7%). Thus, it is not unlikely that the spins undergoing spin-glass-like freezing are the same spins responsible for the Curie-tail susceptibility. We now turn to the *C*1(*T*) term, which is calculated using the two different Schottky approximations mentioned above and plotted in the lower inset of Fig. 7. We note that, below 8 K, *C*_*1*_(*T*) $\propto$*T*^*α*^, where *α* = 0.69 and 0.57 for the *J*_*eff*_ = $\frac{3}{2}$ and $\frac{1}{2}$ Schottky analyses respectively. Such a sublinear form cannot persist down to the lowest temperatures, and importantly is clearly distinct from the phonon contribution visible above 8 K. At the same time, it appears that, among the 70$\%$ of Os ions that are in a +5 state (within an ionic picture) and thus possibly magnetic, less than 15$\%$ are accounted for in either susceptibility or field-dependent specific heat. If the sublinear low-T contribution is due to these unaccounted for, but nevertheless magnetic, Os(+5) ions, then they must be in a type of singlet state due to exchange interactions with a strength greater than the Zeeman energy of an 8 Tesla field, but of a type that invalidates the effective moment approximation below room temperature.

### Electronic structure

Early model treatments of 5d oxides on honeycomb lattices built on the ionic description of crystal field splitting, additionally with strong SOC among orbitals, focused attention on the *t*_*2g*_ subshell *J*_*eff*_ = $\frac{1}{2}$ and $\frac{3}{2}$ subspaces. The relevant energy scales of individual bandwidth W, Hubbard U, Hund’s JH, and SOC strength *ξ*, all lie in the 0.5–1.5 eV range, and studies of the electronic structure and especially the exchange coupling have concluded that the ionic picture provides a challenging starting point at best. The electronic structure of octahedral osmates is complicated by several features. First, the active *t*_*2g*_ orbitals are strongly hybridized with the oxygen 2p orbitals, resulting in strongly coupled Os 5d − O 2p states as the fundamental chemical unit. Osmates in the Ba_2_NaOsO_6_ family, for example, have half of the spin density residing on the O octahedron^38,39^. Iridates behave similarly, leading to their characterization as *molecular orbital compounds*, which can lead to longer range exchange coupling parameters compared to more localized moments^40^. Second, SOC also affects the electronic structure, creating both single-ion as well as exchange anisotropy, the relative effects of which are difficult to disentangle. Third, the distortion from ideal rhombohedral symmetry introduces new lower symmetry Fourier components of the potential that causes band anti-crossings. These in turn result in very narrow, 0.3 eV, individual bandwidths and the likelihood of small gaps, as shown below.

Several theoretical studies of honeycomb iridates have concluded that magnetic interactions beyond the Heisenberg-Kitaev model are important, suggesting a more itinerant picture of the electronic structure^19,41–46^. Many of the general findings for iridates carry over to osmates. Our compound presents the additional complication of Os possessing a nominal valence of +4.7, so one must consider a mixture of d^3^ with d^4^ ions. The ionic picture of d^4^ begins with a non-magnetic *J*_*eff*_ = 0 ion and for d^3^ the ionic value is *J*_*eff*_ = $\frac{3}{2}$, and the insulating nature suggests these different ionic states reside on distinct lattice sites, as opposed to the intermediate valence picture. In this ionic picture, the measured ${\mu }_{eff}$ = 0.85 ${\mu }_{B}$ is challenging to account for, as mentioned above, which leads us to consider the general question of moment formation in nearly itinerant systems.

In an effort to reconcile the valence state measurements with the magneto-thermal measurements, we have applied density functional theory (DFT) methods including SOC, correlation effects, and a fixed atomic spin moment method in our study of Li_2_OsO_3_ (see Methods for the description)^47–52^. Without magnetism, SOC is strong enough to provide a pseudogap but no gap, within the Os *t*_*2g*_ bands. This SOC-driven separation is compromised by crystal subfield splittings, bandwidth effects, and anti-crossings arising from structural distortion away from rhombohedral symmetry leaving two inequivalent Os sites. The resulting band structure (not shown) is that of a very narrow, essentially zero (indirect) gap semiconductor. Due to the molecular orbital nature of the *t*_*2g*_ band complex, intra-atomic repulsion effects as treated by the Hubbard U repulsion are ineffective in opening a gap, for reasonable values of U (2 eV or less). Antiferromagnetic order tends to encourage gap opening, producing Os moments of 0.3 ${\mu }_{B}$.

To include in the modeling the effect of the observed Os moment, we have adopted the constrained atomic moment method as implemented in the *abinit* code^47^. This method proceeds not by specifying a value for U for the Os 5d orbitals, but by fixing the spin moment by applying an intra-atomic Zeeman field determined self-consistently; both magnitude and direction can be specified separately for any atom. Magnitudes of 0.8 ${\mu }_{B}$ and 0.5 ${\mu }_{B}$ have been studied; the latter value represents the ordered component expected of a 0.8 ${\mu }_{B}$ local (C-W) moment. Bandgaps of 0.25 eV and larger were obtained, depending weakly on the imposed moment but strongly on the magnetic alignment (the larger ones were for AFM order). The resulting orbital moments are minor, only a few hundredths of 1 ${\mu }_{B}$, independent of the chosen direction of spin. These results do not fit that of a Mott insulator: there is no robust local moment, and Hubbard U is not needed to open a gap and has little effect on the size of the gap. Thus our model rationalizes the observations of a narrow transport gap and small magnetic moment in Li_2. 15_Os_0.85_O_3_.

**Figure 8:**
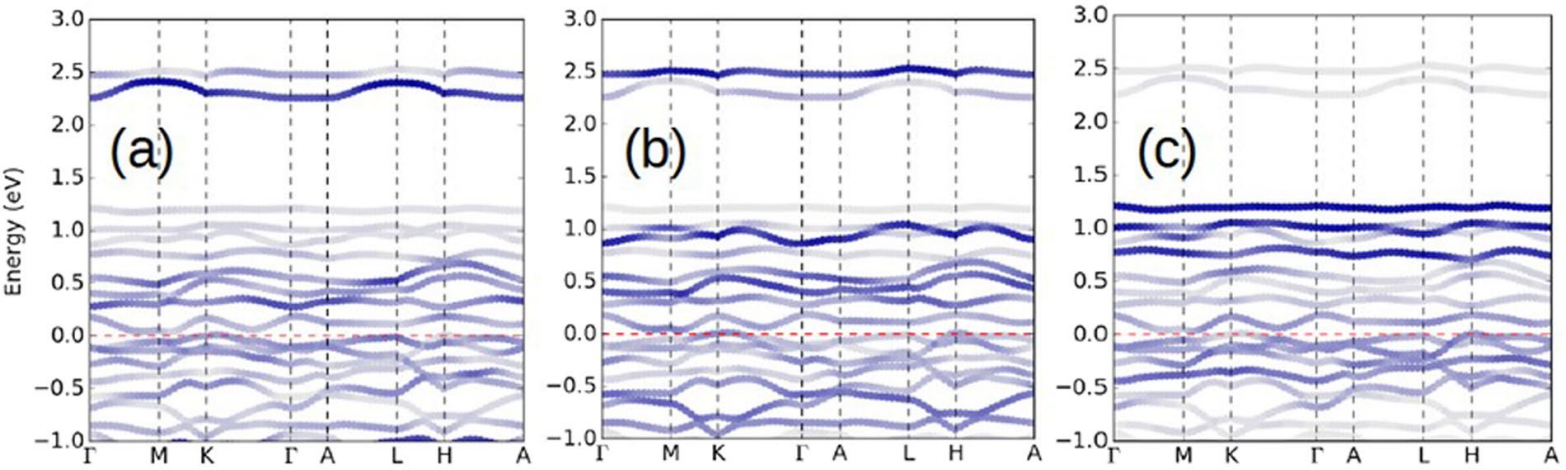
Plot of bands in the Os *t*_*2g*_ region of 25$\%$ Li-substituted Li_2_OsO_3_ on the Os honeycomb sublattice, with energy zero (horizontal red dashed line) denoting the gap region. The fatbands plots emphasize the Os 5d character on (**a**) the Os[0] site with no Li neighbor, (**b**) the Os[1] site with one Li neighbor, and (**c**) the Os[2] site with two Li neighbors. While Os[0] and Os[1] show some differences, Os[2] is qualitatively different.

Due to intermixing of Li on the Os honeycomb lattice, we have made an initial study of the effect of intermixing, by replacing 25$\%$ of the Os sublattice by Li while keeping the Li sublattice intact. The Os moments are fixed with magnitude 0.8 ${\mu }_{B}$ and oriented along separate (111) axes to mimic disordered moments. The resulting band structure, shown in Fig. 8, illustrates the flat individual bands that arise, and that a very small gap exists or is imminent, depending on details of the calculations. The bands are not significantly different in appearance from those of Li_2_OsO_3_.

We now focus on the effect of Li substitution on the remaining Os ions. This 25$\%$ replacement results in three Os sites, denoted by Os[j] which has j Li neighbors, j = 0, 1 or 2. The formal valences of Os in this Li_2_(Li_1/4_Os_3/4_)O_3_ structure should average to 5+. The periodicity leaves Os[0]-Os[1] chains and comparatively isolated Os[2] ions, which in addition to two Li neighbors the Os neighbor is at a long Os-Os separation. The spectral distribution of Os[2] in Fig. 8 is substantially different from the other two, more representative of a lower oxidation state. While simple electron count indicates that Li substitution must oxidize some Os ions, having two Li neighbors strongly affects the formal valence of the Os ion.

This modeling illustrates that the Os valence is sensitive not only to the total charge available, but also to the local environment. Different valence states carry different moments, and sensitivity to the local environment suggests that variation of exchange constants promotes a frustration of magnetic order. Note in Fig. 8 that the spectral distributions of 5d weight are significantly different for Os[0] and Os[1], while that of Os[2] is less weight in the occupied bands. This implication then is that of one Os(4+) ions and two Os(5+) ion, with an average valence of 4.67 consistent with spectroscopic evidence on our samples. More of a specific nature cannot be concluded because the Os moments were constrained (to be equal), whereas those of different valence states would not be equal.

## Summary

We have presented the first example of Os on a honeycomb structure, Li_2.15(3)_Os_0.85(3)_O_3_, and have characterized it with atomic, structural, and magneto-thermal probes. The Os ions have an average valence state of +4.7 and large site disorder exists in the honeycomb layers. This compound is a narrow band gap semiconductor. The magnetic susceptibility and specific heat present a picture in which the effective Os moment is reduced to a value well below that expected from the valence-state measurements, which suggests that the valence electrons are on the verge of itineracy, a conclusion supported by our density functional theory calculations. These results strongly suggest that spin orbit coupling of Os is playing an important role in the collective electronic behavior of this honeycomb system, and that further studies of osmates on frustrating lattices are warranted.

## Methods

### Synthesis and Structure Characterization

Stoichiometric amounts of Li_2_CO_3_ and OsO_2_ (synthesized from osmium metal) were intimately ground, pressed into a pellet, loaded into an alumina crucible, and fired in a tube furnace at 700 C under Argon flow. Firing was repeated to 850 C with 50 C increments, grinding the sample before each firing. Each firing was performed under argon flow. Phase analysis of the powder samples was performed by X-ray diffraction using a Rigaku MiniFlex II diffractometer with Cu K$\alpha$ radiation and a graphite monochromator for the diffracted beam. Time of Flight (TOF) neutron diffraction measurements were collected at ORNL NOMAD BL-1B SNS beamline. X-ray absorption spectroscopy measurements were carried out at beamline 4-ID-D of the Advanced Photon Source at Argonne National Laboratory using a transmission geometry. Reference samples for valence determination included Os metal, Os^4+^O_2_ and Sr_2_FeOs^5+^O_6_^53^.

### Electronic and Thermal Properties

The Seebeck coefficient and electrical conductivity data (350 K–600 K) were collected on an ULVAC ZEM-3 under a helium atmosphere. Magnetization measurements (2 K–300 K) were obtained with a Quantum Design MPMS. Resistivity, $\rho$(*T*), (200 K–350 K) and specific heat, *C(T)*, data (2 K–30 K) were obtained using a Quantum Design PPMS. Magnetic ac-susceptibility data down to 0.1 K were obtained in a ^3^He-^4^He dilution refrigerator with thermal contact to the mixing chamber made via a copper wire bundle bonded to the sample with Stycast 1266 epoxy. Data were obtained at 143 Hz and with an excitation current low enough to eliminate heating from the coils.

### Theoretical Methods

We use the open-source package ABINIT^48^ to perform electronic calculations, with the generalized gradient approximation (GGA)^49^ for the semilocal exchange-correlation functional and the projector augmented wave method (PAW)^50^ for core electrons. A Hubbard U repulsive interaction was applied with magnitude as indicated, Hund’s JH = 0.4 eV was applied, on the Os 5d orbitals^51^. A mesh of 9 × 5 × 9 was used for k-sampling and 500 eV for energy cutoff. The constrained atomic spin moment on Os method^52^ was used in some calculations to fix moments at or near the observed value. Constraints are managed by the use of Lagrangian multipliers, imposed with constraint parameters; the input parameter *λ* = 1.0 was used^48^.

## Electronic supplementary material

Supplementary Information
