# Supplementary material for: Local Moment Instability of Os in Honeycomb Li2.15Os0.85O3
Source: Sci Rep. 2018 Apr 26;8:6605. doi: 10.1038/s41598-018-25028-0 (PMC5919924; doi:10.1038/s41598-018-25028-0)
Supplement: Supplementary file 1 — Supplementary Information [file 41598_2018_25028_MOESM1_ESM.pdf]

Supplementary information for:

Local Moment Instability of Os in Honeycomb $Li_{2.15}Os_{0.85}O_3$

AUTHORS:

M. K. Wallace[1], P. G. LaBarre[2], Jun Li[1], S.-T. Pi[3], W. E. Pickett[3], D.S. Dessau[4], D. Haskel[5], A. P. Ramirez[2], M. A. Subramanian[1*]

AFFILIATIONS:

[1] Department of Chemistry, Oregon State University, Corvallis, OR 97331

[2] Department of Physics, University of California, Santa Cruz, Santa Cruz, CA 95064

[3] Department of Physics, University of California Davis, Davis, CA 95616

[4] Department of Physics, University of Colorado, CO 80309

[5] Advanced Photon Source, Argonne National Laboratory, Argonne IL 60439

*corresponding author: mas.subramanian@oregonstate.edu

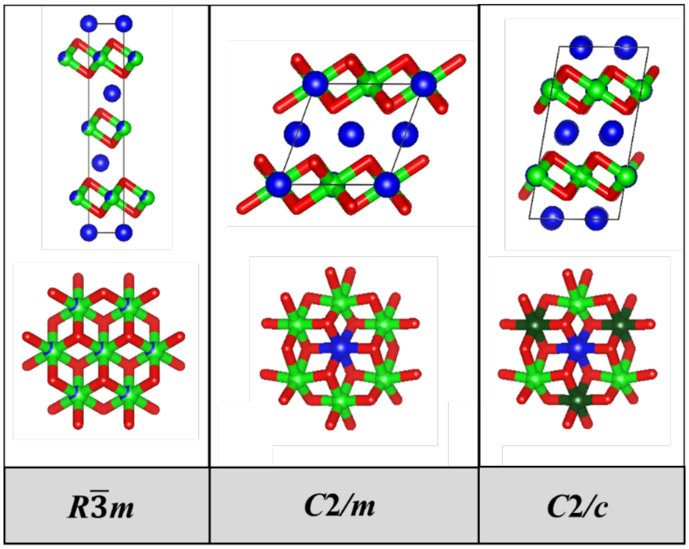

Figure S1. Li_2MO_3 $R\bar{3}m$, $C2/m$, and $C2/c$ unit cells illustrated down the b-axis (top) and corresponding portion of the Li-M layer representing a single hexagon configuration (bottom) with lithium and metal occupying their ideal Wyckoff positions (lithium and metal labeled blue and green respectively). For $C2/c$ there are two unique atomic positions to describe M sites within the LiM_2 layer, represented as the two different shades of green.

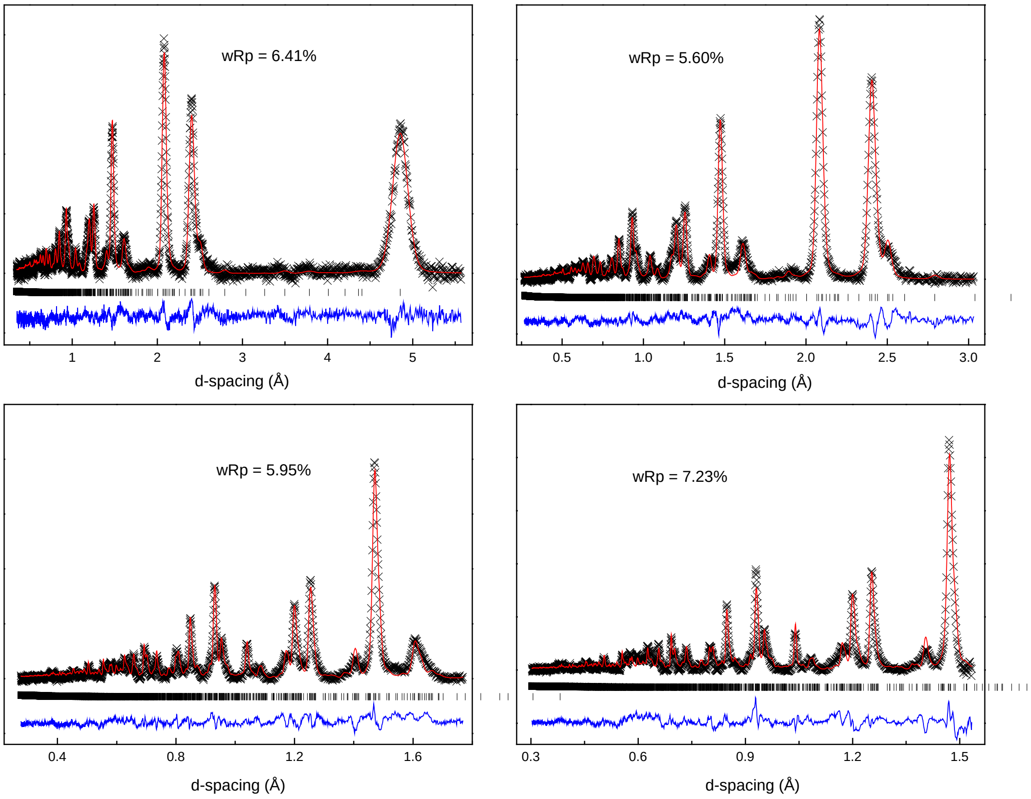

Figure S2. Rietveld refinement of TOF Neutron (Oak Ridge NOMAD BL-1B) diffraction data. The collected data (black cross), Rietveld refinement (red line), and difference (blue line) are presented for each of the four collected banks. Resulting $wR_p = 6.4\%$.

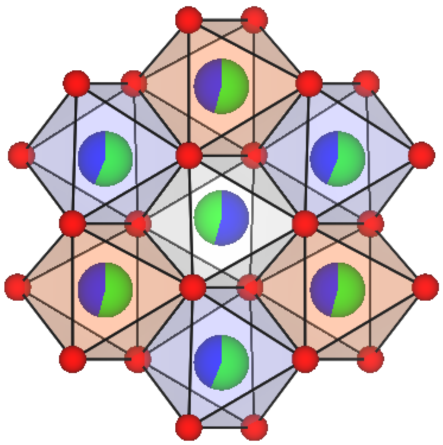

Figure S3. Illustration of the *C2/c* honeycomb ring with lithium and osmium sites labeled according to Table 1 with Os3/Li3, Os4/Li4, and Os5/Li5 octahedral sites as grey, purple, and orange (osmium and lithium labeled green and blue respectively).

Table S1

Bond lengths (Å) and angles (deg)			
Li1 - O1	2.106(9)	Os3(Li3) - O1 x 2	2.025(4)
Li1 - O1'	2.136(9)	Os3(Li3) - O2 x 2	2.046(4)
Li1 - O2	2.187(9)	Os3(Li3) - O3 x 2	2.047(4)
Li1 - O2'	2.110(9)		
Li1 - O3	2.138(9)	Os4(Li4) - O1 x 2	2.020(4)
Li1 - O3'	2.188(9)	Os4(Li4) - O2 x 2	2.023(4)
		Os4(Li4) - O3 x 2	2.014(4)
Li2 - O1 x 2	2.175(4)		
Li2 - O2 x 2	2.220(4)	Os5(Li5) - O1 x 2	2.005(4)
Li2 - O3 x 2	2.113(4)	Os5(Li5) - O2 x 2	2.035(4)
		Os5(Li5) - O3 x 2	2.010(4)
Os3(Li3) - Os4(Li4) x 2	2.978(5)		
Os3(Li3) - Os5(Li5) x 2	2.866(4)	O1 - Os3(Li3) - O2	90.50(2)
Os3(Li3) - Os5(Li5)	3.088(9)	O2 - Os3(Li3) - O3	175.92(3)
		O1 - Os4(Li4) - O2	94.46(2)
		O2 - Os4(Li4) - O3	178.67(3)
		O1 - Os5(Li5) - O2	94.15(2)
		O2 - Os5(Li5) - O3	176.06(3)

Stacking Fault Modeling

The DIFFaX program requires the cell axes to be defined with the c-axis perpendicular to the fault direction. For a monoclinic space group a new idealized unit cell for each individual layer must therefore be established. The new hexagonal unit cell was defined as $a_{hex} = a_{C2/c}$ and $c_{hex} = \left[\left(c_{C2/c} \times \cos\left(\beta_{C2/c} - 90\right)\right)/2\right](z)$, with z as the number of hexagonal layers used for the model. Division of 2 is based on the number of repeating Li-LiM$_2$ layers within *C*2/*c* unit cell. Layer extension in a- and b- direction was set to infinite. The single layer used in DIFFaX and Li-LiOs$_2$ *C*2/*c* layer are illustrated in Fig S4. To replicate the ideal stacking sequence of Li_2OsO_3 (*C2/c*), layer sequencing was characterized by $[1/3,0,1/z]$ transition vector (R_1). To replicate stacking fault contribution, transition vectors $[0,1/3,1/z]$ and $[2/3,0,1/z]$ (R_2 and R_3) were incorporated. Probabilities were assigned to each of the three transition vectors, with $\alpha_1 + \alpha_2 + \alpha_3 = 1$ for each layer. For the latter two transition vectors, there was no tangible difference in the model when varying the contribution for a given total stacking fault probability. Thus for the models presented, the assumption $\alpha_2 = \alpha_3$ was implemented. Fig S5 shows a comparison of the measured XRD pattern (top black line), along with 0% site disorder | 0% stacking fault, 0% site disorder | 30% stacking fault, and 50% site disorder | 0% stacking fault simulated patterns (blue lines) within 17-35 2θ region. With incorporation of stacking faults, only (002) and (020) peaks within 17 – 35 2θ range do not broaden (Fig S6). Similar observations for other DIFFaX modeled Li_2MO_3 systems exist [S1, S2, S3]. Calculated XRD patterns with site disorder equivalent to Table 1 without and with 10% stacking faults are shown in Fig S7. The absence of the (020) peak is therefore attributed to Li-Os site disorder within the LiOs$_2$ layers.

References for SI section

S1. Wallace, D. C. & McQueen, T. M. New honeycomb iridium(v) oxides: $NaIrO_3$ and $Sr_3CaIr_2O_9$. *Dalton Trans* **44**, 20344, (2015).

S2. Wallace, D. C., Brown, C. M. & McQueen, T. M. Evolution of magnetism in $Na_{1-d}(Na_{1-x}Mg_x)Ir_2O_6$ the series of honeycomb iridates. *J. Solid State Chem.* **224**, 28–35 (2015).

S3. Bette, S. et al. Solution of the heavily stacking faulted crystal structure of the honeycomb iridate $H_3LiIr_2O_6$. *Dalton Trans* **46**, 15216 (2017).

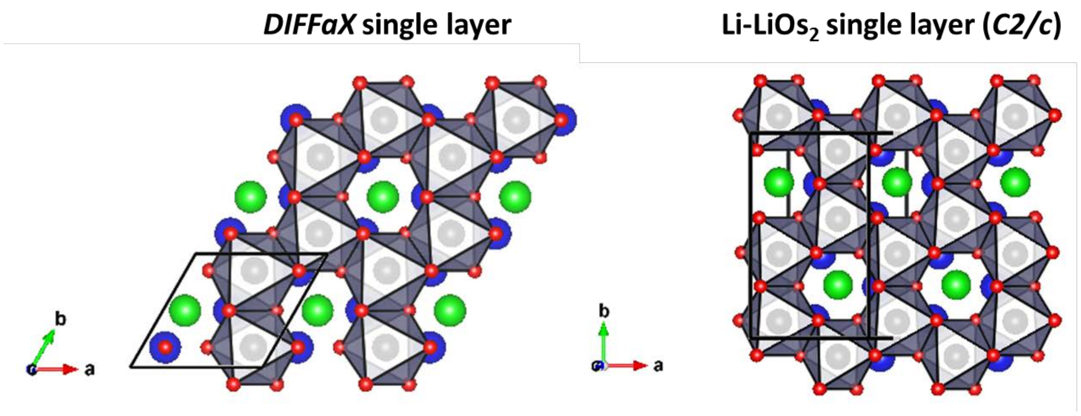

Figure S4. Illustration of the DIFFaX single layer and Li-$LiOs_2$ single layer (*C*2/*c*). Osmium and oxygen are represented as black and red spheres, respectively. Lithium residing within the honeycomb rings are represented as green spheres and lithium beneath the honeycomb layers as blue spheres.

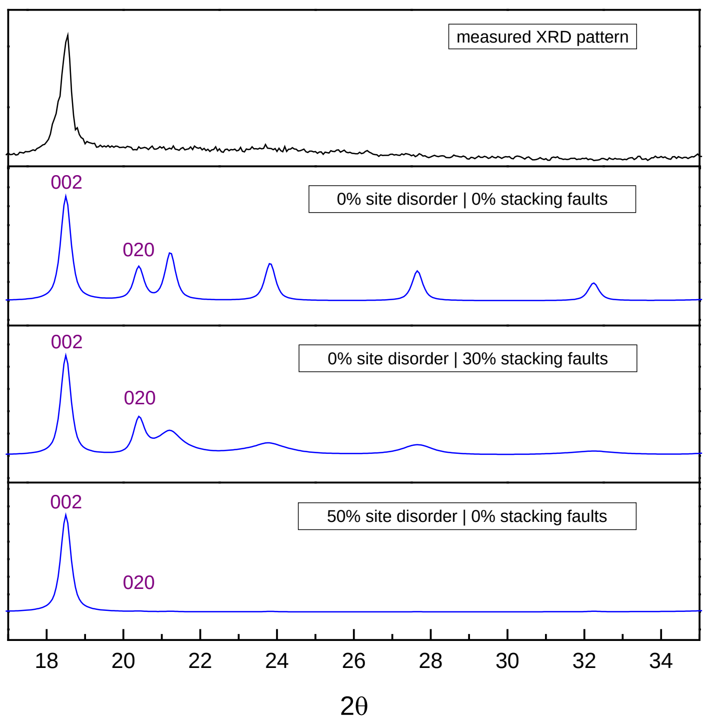

Figure S5. Measured XRD-pattern (black) and simulated DIFFaX patterns of stacking faulted without site disorder and 50% site disorder of Li_2OsO_3 within 17 – 35 2θ range.

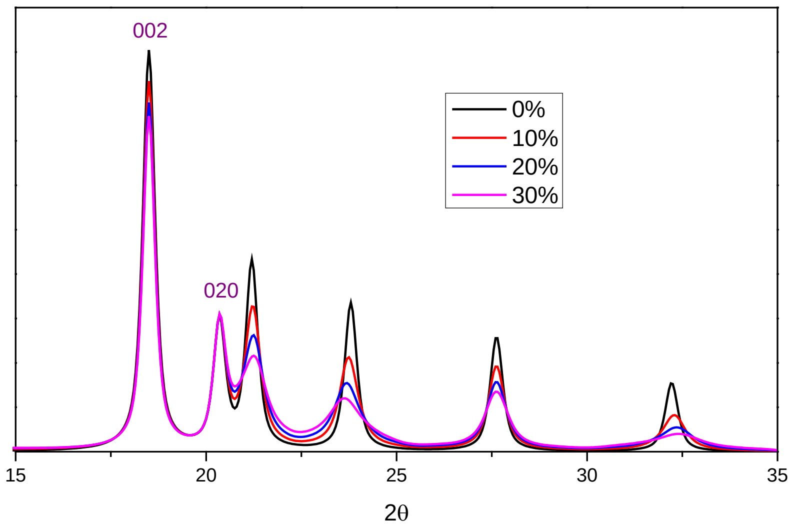

Figure S6. Simulated DIFFaX XRD patterns with 0% - 30% staking faults and no site disorder within 15 – 35 2θ range
